# Picric acid–2,4,6-trichloro­aniline (1/1)

**DOI:** 10.1107/S160053681100571X

**Published:** 2011-03-12

**Authors:** Wan-Qiang Wang

**Affiliations:** a Department of Chemistry and Biology, Xiangfan University, Xiangfan 441053, People’s Republic of China

## Abstract

In the title adduct, C_6_H_4_Cl_3_N·C_6_H_3_N_3_O_7_, the two benzene rings are almost coplanar, with a dihedral angle of 1.19 (1)° and an inter-ring centroid–centroid separation of 4.816 (2) Å. The crystal structure is stabilized by inter­molecular N—H⋯O_nitro_ hydrogen bonds, giving a chain structure. In addition, there are phenol–nitro O—H⋯O inter­actions.

## Related literature

The crystal structures of picrate salts and picric acid complexes have been studied to investigate charge-transfer processes, see: Nagata *et al.* (1995 ▶); Smith *et al.* (2004 ▶). For the crystal structures of picric acid complexes, see: Li (2009 ▶); Sivaramkumar *et al.* (2010 ▶).
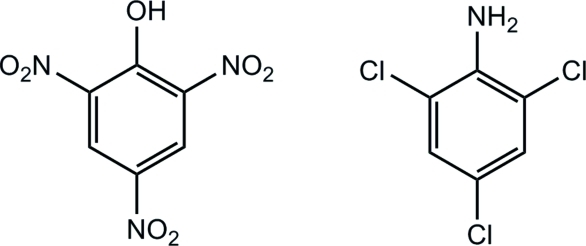


## Experimental

### 

#### Crystal data

C_6_H_4_Cl_3_N·C_6_H_3_N_3_O_7_
*M* *_r_* = 425.57
Orthorhombic, 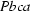

*a* = 9.2162 (14) Å
*b* = 10.0174 (14) Å
*c* = 35.051 (5) Å
*V* = 3236.0 (8) Å^3^
*Z* = 8
Mo *K*α radiation
μ = 0.61 mm^−1^
*T* = 298 K
0.16 × 0.12 × 0.10 mm

#### Data collection

Bruker SMART CCD area-detector diffractometer
Absorption correction: multi-scan (*SADABS*; Sheldrick, 1997 ▶) *T*_min_ = 0.908, *T*_max_ = 0.941
19589 measured reflections
3186 independent reflections
2287 reflections with *I* > 2σ(*I*)
*R*_int_ = 0.076

#### Refinement

*R*[*F* ^2^ > 2σ(*F* ^2^)] = 0.058
*wR*(*F* ^2^) = 0.120
*S* = 1.10
3186 reflections
244 parameters
2 restraints
H atoms treated by a mixture of independent and constrained refinement
Δρ_max_ = 0.21 e Å^−3^
Δρ_min_ = −0.27 e Å^−3^

### 

Data collection: *SMART* (Bruker, 2001 ▶); cell refinement: *SAINT* (Bruker, 1999 ▶); data reduction: *SAINT*; program(s) used to solve structure: *SHELXS97* (Sheldrick, 2008 ▶); program(s) used to refine structure: *SHELXL97* (Sheldrick, 2008 ▶); molecular graphics: *SHELXTL* (Sheldrick, 2008 ▶); software used to prepare material for publication: *SHELXTL*.

## Supplementary Material

Crystal structure: contains datablocks I, global. DOI: 10.1107/S160053681100571X/zs2097sup1.cif

Structure factors: contains datablocks I. DOI: 10.1107/S160053681100571X/zs2097Isup2.hkl

Additional supplementary materials:  crystallographic information; 3D view; checkCIF report

## Figures and Tables

**Table 1:** Hydrogen-bond geometry (Å, °)

*D*—H⋯*A*	*D*—H	H⋯*A*	*D*⋯*A*	*D*—H⋯*A*
O1—H1*A*⋯O7	0.89 (4)	1.76 (4)	2.546 (4)	145 (4)
N4—H4*A*⋯O5^i^	0.85 (2)	2.39 (2)	3.159 (4)	150 (3)
N4—H4*B*⋯O6^ii^	0.84 (2)	2.40 (2)	3.194 (4)	156 (4)

Symmetry codes: (i) 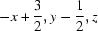; (ii) 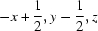.

**Fig. 1.**
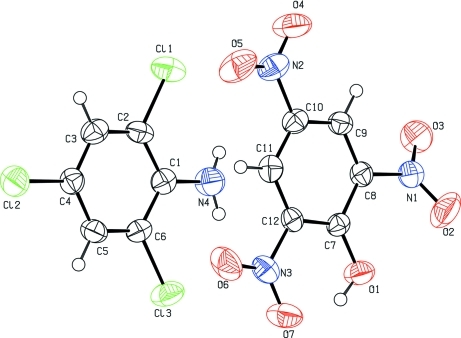
The title compound with the atom-numbering scheme, with displacement ellipsoids drawn at the 30% probability level.

## supplementary crystallographic information

### Comment

2,4,6-Trinitrophenol (picric acid), was primarily used to manufacture explosives and is also used as an intermediate in dye manufacturing. The crystal structures of a large number of picrate salts and picric acid complexes have been studied to determine the conformational features and to understand charge transfer processes (Li *et al.*, 2009; Nagata *et al.*, 1995; Sivaramkumar *et al.*, 2010, Smith *et al.*, 2004). We herein report the 1:1 cocrystal structure of 2,4,6-trichloroaniline and picric acid C_6_H_4_Cl_3_N . C_6_H_3_N_3_O_7_ (I) (Fig. 1). In the title adduct, the two phenyl rings are almost coplanar with a dihedral angle of 1.19 (1)° and an inter-ring centroid separation of 4.816 (2) Å. The crystal structure is stabilized by intermolecular N—H···O_nitro_ hydrogen bonds giving a one-dimensional chain structure and in addition, intramolecular N—H···Cl and phenol O—H···O(nitro) interactions are observed (Table 1).

### Experimental

2,4,6-Trichloroaniline (0.19 g, 1.0 mmol) and picric acid (0.23 g, 1.0 mmol) were dissolved in MeOH-CH_2_Cl_2_ (3:1) and the mixture was kept at room temperature for one week. Red crystals suitable for single-crystal X-ray diffraction were obtained.

### Refinement

The O– and N-bound H atoms were located in a difference map and refined isotropically. The remaining H atoms were positioned geometrically (C—H = 0.93 Å) and allowed to ride on their parent atoms, with *U*_iso_(H) = 1.2*U*_eq_(C).

### Crystal data

**Table:**

C_6_H_4_Cl_3_N·C_6_H_3_N_3_O_7_	*F*(000) = 1712
*M**_r_* = 425.57	*D*_x_ = 1.747 Mg m^−^^3^
Orthorhombic, *P**b**c**a*	Mo *K*α radiation, λ = 0.71073 Å
Hall symbol: -P 2ac 2ab	Cell parameters from 1781 reflections
*a* = 9.2162 (14) Å	θ = 2.5–19.2°
*b* = 10.0174 (14) Å	µ = 0.61 mm^−^^1^
*c* = 35.051 (5) Å	*T* = 298 K
*V* = 3236.0 (8) Å^3^	Block, red
*Z* = 8	0.16 × 0.12 × 0.10 mm

### Data collection

**Table:**

Bruker SMART CCD area-detector diffractometer	3186 independent reflections
Radiation source: fine-focus sealed tube	2287 reflections with *I* > 2σ(*I*)
graphite	*R*_int_ = 0.076
φ and ω scans	θ_max_ = 26.0°, θ_min_ = 2.3°
Absorption correction: multi-scan (*SADABS*; Sheldrick, 1997)	*h* = −11→11
*T*_min_ = 0.908, *T*_max_ = 0.941	*k* = −12→10
19589 measured reflections	*l* = −41→43

### Refinement

**Table:**

Refinement on *F*^2^	Primary atom site location: structure-invariant direct methods
Least-squares matrix: full	Secondary atom site location: difference Fourier map
*R*[*F*^2^ > 2σ(*F*^2^)] = 0.058	Hydrogen site location: inferred from neighbouring sites
*wR*(*F*^2^) = 0.120	H atoms treated by a mixture of independent and constrained refinement
*S* = 1.10	*w* = 1/[σ^2^(*F*_o_^2^) + (0.0381*P*)^2^ + 1.3007*P*] where *P* = (*F*_o_^2^ + 2*F*_c_^2^)/3
3186 reflections	(Δ/σ)_max_ = 0.001
244 parameters	Δρ_max_ = 0.21 e Å^−^^3^
2 restraints	Δρ_min_ = −0.27 e Å^−^^3^

### Special details

**Table:**

Geometry. All e.s.d.'s (except the e.s.d. in the dihedral angle between two l.s. planes) are estimated using the full covariance matrix. The cell e.s.d.'s are taken into account individually in the estimation of e.s.d.'s in distances, angles and torsion angles; correlations between e.s.d.'s in cell parameters are only used when they are defined by crystal symmetry. An approximate (isotropic) treatment of cell e.s.d.'s is used for estimating e.s.d.'s involving l.s. planes.
Refinement. Refinement of *F*^2^ against ALL reflections. The weighted *R*-factor *wR* and goodness of fit *S* are based on *F*^2^, conventional *R*-factors *R* are based on *F*, with *F* set to zero for negative *F*^2^. The threshold expression of *F*^2^ > σ(*F*^2^) is used only for calculating *R*-factors(gt) *etc*. and is not relevant to the choice of reflections for refinement. *R*-factors based on *F*^2^ are statistically about twice as large as those based on *F*, and *R*- factors based on ALL data will be even larger.

### Fractional atomic coordinates and isotropic or equivalent isotropic displacement parameters (Å^2^)

**Table:**

	*x*	*y*	*z*	*U*_iso_*/*U*_eq_	
C1	0.4507 (4)	0.4557 (3)	0.57639 (9)	0.0373 (8)	
C2	0.5792 (3)	0.4955 (3)	0.55888 (10)	0.0396 (8)	
C3	0.5881 (4)	0.6056 (4)	0.53560 (10)	0.0422 (9)	
H3	0.6760	0.6295	0.5245	0.051*	
C4	0.4651 (4)	0.6801 (3)	0.52893 (9)	0.0390 (8)	
C5	0.3352 (4)	0.6456 (3)	0.54541 (10)	0.0415 (9)	
H5	0.2523	0.6960	0.5408	0.050*	
C6	0.3293 (3)	0.5361 (3)	0.56874 (9)	0.0375 (8)	
C7	0.4085 (3)	0.4501 (3)	0.70238 (9)	0.0325 (7)	
C8	0.5325 (3)	0.3724 (3)	0.70914 (9)	0.0343 (8)	
C9	0.6640 (3)	0.3992 (3)	0.69253 (9)	0.0360 (8)	
H9	0.7437	0.3447	0.6972	0.043*	
C10	0.6760 (4)	0.5082 (3)	0.66889 (9)	0.0379 (8)	
C11	0.5597 (4)	0.5899 (3)	0.66136 (9)	0.0379 (8)	
H11	0.5694	0.6642	0.6456	0.046*	
C12	0.4278 (3)	0.5584 (3)	0.67784 (9)	0.0324 (8)	
Cl1	0.73498 (10)	0.40282 (11)	0.56753 (3)	0.0607 (3)	
Cl2	0.47396 (11)	0.81912 (11)	0.49911 (3)	0.0592 (3)	
Cl3	0.16542 (10)	0.49502 (10)	0.59030 (3)	0.0560 (3)	
N1	0.5268 (3)	0.2597 (3)	0.73550 (9)	0.0478 (8)	
N2	0.8171 (3)	0.5390 (4)	0.65186 (9)	0.0507 (8)	
N3	0.3064 (3)	0.6474 (3)	0.66942 (9)	0.0452 (8)	
N4	0.4439 (4)	0.3486 (3)	0.60078 (10)	0.0520 (8)	
H4A	0.512 (3)	0.290 (3)	0.6009 (11)	0.062*	
H4B	0.359 (2)	0.332 (4)	0.6079 (11)	0.062*	
O1	0.2826 (3)	0.4170 (2)	0.71812 (7)	0.0494 (7)	
H1A	0.218 (5)	0.479 (4)	0.7114 (11)	0.074*	
O2	0.4487 (4)	0.2669 (3)	0.76291 (9)	0.0979 (13)	
O3	0.6055 (3)	0.1642 (3)	0.72928 (8)	0.0643 (8)	
O4	0.9144 (3)	0.4573 (3)	0.65587 (9)	0.0711 (9)	
O5	0.8294 (3)	0.6445 (3)	0.63491 (9)	0.0726 (9)	
O6	0.3275 (3)	0.7422 (3)	0.64860 (8)	0.0672 (8)	
O7	0.1878 (3)	0.6247 (3)	0.68450 (8)	0.0602 (8)	

### Atomic displacement parameters (Å^2^)

**Table:**

	*U*^11^	*U*^22^	*U*^33^	*U*^12^	*U*^13^	*U*^23^
C1	0.041 (2)	0.035 (2)	0.0352 (19)	−0.0034 (16)	0.0003 (15)	−0.0064 (15)
C2	0.0318 (19)	0.042 (2)	0.045 (2)	0.0053 (16)	−0.0046 (15)	−0.0061 (17)
C3	0.0369 (19)	0.045 (2)	0.045 (2)	−0.0084 (17)	0.0048 (16)	−0.0036 (17)
C4	0.0398 (19)	0.037 (2)	0.040 (2)	−0.0048 (17)	−0.0010 (15)	0.0042 (16)
C5	0.0319 (18)	0.045 (2)	0.047 (2)	0.0040 (16)	−0.0042 (16)	0.0025 (17)
C6	0.0333 (18)	0.040 (2)	0.0395 (19)	−0.0019 (16)	0.0051 (15)	−0.0023 (16)
C7	0.0329 (18)	0.0345 (19)	0.0299 (17)	−0.0024 (15)	0.0016 (14)	−0.0052 (14)
C8	0.0383 (18)	0.0281 (19)	0.0365 (18)	0.0010 (15)	0.0009 (15)	0.0055 (14)
C9	0.0330 (17)	0.037 (2)	0.0377 (18)	0.0034 (15)	−0.0012 (15)	−0.0017 (15)
C10	0.0355 (19)	0.039 (2)	0.0392 (19)	−0.0075 (16)	0.0032 (15)	−0.0038 (16)
C11	0.044 (2)	0.032 (2)	0.0385 (19)	−0.0068 (16)	−0.0021 (15)	0.0007 (15)
C12	0.0355 (18)	0.0322 (19)	0.0296 (17)	−0.0002 (15)	−0.0040 (14)	−0.0033 (14)
Cl1	0.0420 (5)	0.0648 (7)	0.0754 (7)	0.0154 (5)	−0.0015 (5)	−0.0019 (5)
Cl2	0.0527 (6)	0.0609 (7)	0.0641 (6)	−0.0095 (5)	−0.0010 (5)	0.0236 (5)
Cl3	0.0402 (5)	0.0565 (6)	0.0712 (7)	−0.0038 (5)	0.0176 (5)	0.0094 (5)
N1	0.0449 (18)	0.050 (2)	0.0481 (19)	0.0053 (16)	0.0021 (15)	0.0120 (15)
N2	0.0446 (19)	0.052 (2)	0.056 (2)	−0.0106 (17)	0.0097 (16)	−0.0038 (17)
N3	0.0457 (19)	0.041 (2)	0.0492 (19)	0.0058 (15)	−0.0081 (15)	0.0014 (15)
N4	0.049 (2)	0.045 (2)	0.062 (2)	0.0031 (16)	0.0048 (18)	0.0101 (17)
O1	0.0370 (14)	0.0485 (17)	0.0626 (17)	0.0027 (12)	0.0115 (12)	0.0103 (13)
O2	0.105 (3)	0.114 (3)	0.075 (2)	0.052 (2)	0.051 (2)	0.0556 (19)
O3	0.0714 (19)	0.0479 (18)	0.073 (2)	0.0191 (15)	0.0092 (15)	0.0161 (14)
O4	0.0390 (16)	0.079 (2)	0.095 (2)	0.0047 (16)	0.0162 (15)	0.0066 (18)
O5	0.070 (2)	0.0587 (19)	0.089 (2)	−0.0161 (16)	0.0300 (16)	0.0133 (17)
O6	0.0654 (19)	0.0548 (18)	0.081 (2)	0.0084 (15)	−0.0129 (15)	0.0285 (16)
O7	0.0393 (15)	0.0554 (18)	0.086 (2)	0.0101 (13)	0.0032 (14)	0.0066 (15)

### Geometric parameters (Å, °)

**Table:**

C1—N4	1.373 (4)	C9—C10	1.374 (4)
C1—C2	1.392 (5)	C9—H9	0.9300
C1—C6	1.404 (5)	C10—C11	1.375 (5)
C2—C3	1.374 (5)	C10—N2	1.464 (4)
C2—Cl1	1.736 (3)	C11—C12	1.382 (4)
C3—C4	1.377 (5)	C11—H11	0.9300
C3—H3	0.9300	C12—N3	1.461 (4)
C4—C5	1.373 (4)	N1—O2	1.202 (4)
C4—Cl2	1.743 (3)	N1—O3	1.221 (4)
C5—C6	1.370 (4)	N2—O5	1.218 (4)
C5—H5	0.9300	N2—O4	1.222 (4)
C6—Cl3	1.739 (3)	N3—O6	1.213 (4)
C7—O1	1.327 (4)	N3—O7	1.235 (4)
C7—C12	1.396 (4)	N4—H4A	0.854 (18)
C7—C8	1.404 (4)	N4—H4B	0.842 (18)
C8—C9	1.371 (4)	O1—H1A	0.89 (4)
C8—N1	1.460 (4)		
			
N4—C1—C2	122.5 (3)	C8—C9—H9	120.6
N4—C1—C6	122.0 (3)	C10—C9—H9	120.6
C2—C1—C6	115.4 (3)	C9—C10—C11	121.7 (3)
C3—C2—C1	122.8 (3)	C9—C10—N2	119.0 (3)
C3—C2—Cl1	118.9 (3)	C11—C10—N2	119.3 (3)
C1—C2—Cl1	118.3 (3)	C10—C11—C12	118.0 (3)
C2—C3—C4	119.1 (3)	C10—C11—H11	121.0
C2—C3—H3	120.4	C12—C11—H11	121.0
C4—C3—H3	120.4	C11—C12—C7	123.2 (3)
C5—C4—C3	120.7 (3)	C11—C12—N3	116.7 (3)
C5—C4—Cl2	119.6 (3)	C7—C12—N3	120.0 (3)
C3—C4—Cl2	119.8 (3)	O2—N1—O3	123.0 (3)
C6—C5—C4	119.1 (3)	O2—N1—C8	118.8 (3)
C6—C5—H5	120.4	O3—N1—C8	118.1 (3)
C4—C5—H5	120.4	O5—N2—O4	124.7 (3)
C5—C6—C1	122.8 (3)	O5—N2—C10	117.7 (3)
C5—C6—Cl3	118.9 (3)	O4—N2—C10	117.6 (3)
C1—C6—Cl3	118.3 (3)	O6—N3—O7	122.9 (3)
O1—C7—C12	124.2 (3)	O6—N3—C12	118.4 (3)
O1—C7—C8	120.2 (3)	O7—N3—C12	118.6 (3)
C12—C7—C8	115.5 (3)	C1—N4—H4A	120 (3)
C9—C8—C7	122.6 (3)	C1—N4—H4B	112 (3)
C9—C8—N1	116.9 (3)	H4A—N4—H4B	123 (4)
C7—C8—N1	120.4 (3)	C7—O1—H1A	108 (3)
C8—C9—C10	118.9 (3)		
			
N4—C1—C2—C3	−177.4 (3)	C8—C9—C10—C11	−0.4 (5)
C6—C1—C2—C3	−0.1 (5)	C8—C9—C10—N2	178.5 (3)
N4—C1—C2—Cl1	1.6 (5)	C9—C10—C11—C12	−1.0 (5)
C6—C1—C2—Cl1	179.0 (2)	N2—C10—C11—C12	−180.0 (3)
C1—C2—C3—C4	−0.4 (5)	C10—C11—C12—C7	1.7 (5)
Cl1—C2—C3—C4	−179.4 (3)	C10—C11—C12—N3	179.8 (3)
C2—C3—C4—C5	0.4 (5)	O1—C7—C12—C11	−179.0 (3)
C2—C3—C4—Cl2	−179.4 (3)	C8—C7—C12—C11	−0.8 (5)
C3—C4—C5—C6	0.2 (5)	O1—C7—C12—N3	2.9 (5)
Cl2—C4—C5—C6	179.9 (3)	C8—C7—C12—N3	−178.8 (3)
C4—C5—C6—C1	−0.7 (5)	C9—C8—N1—O2	144.8 (4)
C4—C5—C6—Cl3	178.7 (3)	C7—C8—N1—O2	−33.6 (5)
N4—C1—C6—C5	178.0 (3)	C9—C8—N1—O3	−32.8 (5)
C2—C1—C6—C5	0.6 (5)	C7—C8—N1—O3	148.8 (3)
N4—C1—C6—Cl3	−1.4 (5)	C9—C10—N2—O5	−171.2 (3)
C2—C1—C6—Cl3	−178.8 (2)	C11—C10—N2—O5	7.8 (5)
O1—C7—C8—C9	177.6 (3)	C9—C10—N2—O4	8.4 (5)
C12—C7—C8—C9	−0.7 (5)	C11—C10—N2—O4	−172.6 (3)
O1—C7—C8—N1	−4.1 (5)	C11—C12—N3—O6	0.4 (4)
C12—C7—C8—N1	177.6 (3)	C7—C12—N3—O6	178.5 (3)
C7—C8—C9—C10	1.4 (5)	C11—C12—N3—O7	−178.0 (3)
N1—C8—C9—C10	−177.0 (3)	C7—C12—N3—O7	0.2 (5)

### Hydrogen-bond geometry (Å, °)

**Table:**

*D*—H···*A*	*D*—H	H···*A*	*D*···*A*	*D*—H···*A*
O1—H1A···O7	0.89 (4)	1.76 (4)	2.546 (4)	145 (4)
N4—H4A···Cl1	0.85 (2)	2.62 (4)	2.975 (3)	106 (3)
N4—H4A···O5^i^	0.85 (2)	2.39 (2)	3.159 (4)	150 (3)
N4—H4B···Cl3	0.84 (2)	2.49 (3)	2.979 (3)	118 (3)
N4—H4B···O6^ii^	0.84 (2)	2.40 (2)	3.194 (4)	156 (4)

Symmetry codes: (i) −*x*+3/2, *y*−1/2, *z*; (ii) −*x*+1/2, *y*−1/2, *z*.
